# The Impact of a Health Empowerment Program on Self-Care Enablement and Mental Health among Low-Income Families: Evidence from a 5 Year Cohort Study in Hong Kong

**DOI:** 10.3390/ijerph20065168

**Published:** 2023-03-15

**Authors:** Fangcao Lu, Carlos King Ho Wong, Emily Tsui Yee Tse, Amy Pui Pui Ng, Lanlan Li, Joyce Sau Mei Lam, Laura Bedford, Daniel Yee Tak Fong, Patrick Ip, Cindy Lo Kuen Lam

**Affiliations:** 1 Department of Family Medicine and Primary Care, Li Ka Shing Faculty of Medicine, The University of Hong Kong, Hong Kong 999077, China; 2 Department of Pharmacology and Pharmacy, Li Ka Shing Faculty of Medicine, The University of Hong Kong, Hong Kong 999077, China; 3 Laboratory of Data Discovery for Health (D24H), Hong Kong Science and Technology Park, Hong Kong 999077, China; 4 Department of Family Medicine and Primary Care, The University of Hong Kong-Shenzhen Hospital, Shenzhen 518000, China; 5 School of Nursing, Li Ka Shing Faculty of Medicine, The University of Hong Kong, Hong Kong 999077, China; 6 Department of Paediatrics and Adolescent Medicine, Li Ka Shing Faculty of Medicine, The University of Hong Kong, Hong Kong 999077, China; 7 Department of Paediatrics and Adolescent Medicine, Hong Kong Children’s Hospital, Hong Kong 999077, China

**Keywords:** empowerment, self-care enablement, low-income families, health-related quality of life, mental health

## Abstract

Health empowerment can be an effective way to reduce health inequities. This prospective cohort study evaluated the 5 year impact of a health empowerment program (HEP) on health outcomes among adults from low-income families. The Patient Enablement Instrument version 2 (PEI-2), Depression, Anxiety and Stress Scale 21 (DASS-21), and 12 item Short-Form Health Survey version 2 (SF-12v2) were administered at baseline and follow-up for both intervention and comparison groups. A total of 289 participants (*n* = 162 for intervention group, *n* = 127 for comparison group) were included in the analysis. Most of the participants were female (72.32%), and aged from 26 to 66 years old (M = 41.63, SD = 6.91). Linear regressions weighted by inverse probability weighting using the propensity score showed that, after follow-up of 5 years, the intervention group demonstrated significantly greater increases in all items and total scores for the PEI-2 (all B > 0.59, *p* < 0.001), greater decreases in the DASS depression score (B = −1.98 *p* = 0.001), and greater increases in the Mental Component Summary score of the SF-12v2 (B = 2.99, *p* = 0.027) than the comparison group. The HEP may be an effective intervention enabling adults from low-income families to manage their health-related issues and improve their mental health, as evidenced by our study.

## 1. Introduction

Poverty is a global problem linked to poor health outcomes [1]. In addition to difficulties in accessing healthy food, clean water, and safe shelter, limited healthcare recourses are also a common problem for people from low-income families [2]. Indeed, a study found that people who received low incomes and/or lived in poverty had poorer health than the age–sex-matched individuals from the general population [3]. Moreover, healthcare expenses can further divert already limited disposable income from the educational, social, and other needs of families, hindering children’s development and resulting in trans-generational poverty [4,5,6]. Thus, the close link between poverty and poor health forms a vicious cycle [7]. There have been calls for the development of effective interventions to improve health among people from low-income families and break the cycle of poverty and poorer health [1].

Primary healthcare plays a key role in improving public health and reducing health inequities [8,9]. Starting from self-care, primary healthcare involves health promotion, disease prevention, and management of health conditions [10]. The central component of primary healthcare is health empowerment, a process through which people are motivated to take greater control over their lives and health-related decisions [11]. The concept of health empowerment entails working in partnership with individuals to enhance health literacy, desire, self-assurance, ways of action, and utilization of external resources in order to maintain good health, practice self-care, and increase appropriate use of healthcare services [12].

There is a growing body of literature on the benefits of health empowerment (HE) interventions. First, HE helps improve health-related abilities, such as health literacy [13] and utilization of health services [14]. Second, it can stimulate the adoption of healthy habits, such as increasing physical activity and reducing sedentary behavior [15]. Third, HE can modify health-related attitudes, including enhancing self-efficacy in self-care [16], self-determination [17], and self-efficacy in physical activity [18]. Last, HE has been found to improve physical and psychological health among a range of participant groups, including people with diabetes mellitus [12], adolescents [17], and homebound older adults [19]. The effectiveness of HE has been documented in both high-income (the U.S. [18,19], Italy [20], South Korea [15], Sweden [21], Taiwan [14,16]) and low-income regions (Thailand [13], India [17]). Nevertheless, evidence on the effectiveness of HE for people from relatively low-income families in high-income regions is limited. Most studies targeting this disadvantaged group were conducted in U.S., showing effectiveness in reducing depression in both adults [22,23,24] and children [25,26] while increasing perceived quality of life and positive affect [27]. The HE prevented hospitalizations [28] and pediatric emergency room and clinic visits [29]. HE can also promote healthier lifestyles, such as through assisting in smoking cessation [30], promoting healthy eating, increasing physical activity [31,32], assisting in the adoption of general environmental health precautions, increasing self-efficacy [33], improving problem-solving abilities [34], and improving intellectual academic achievements [35], among members of low-income families.

The HE interventions reported in the literature tend to be highly controlled, unidimensional strategies delivered exclusively to either parents or children in low-income families using short-term outcomes [22,27,28,34], which have limitations in terms of generalizability and sustainability. Building on the existing evidence, we designed a long-term, complex, community-based health empowerment program (HEP) with intercalated components comprising annual health assessments, health talks, self-care enablement courses, and health ambassador training, which were available to both parents and children in their natural environment on a voluntary basis. We believed that such an HE intervention would be more feasible and sustainable for self-care enablement and health. We hope this will stimulate a new direction in HE interventions that may eventually lead to specific changes in health policy and services, reducing the health inequity among people from low-income families.

Hong Kong has undergone rapid economic development since the late 20th century, becoming a high-income region [36]. However, it has one of the highest Gini coefficients in the world (0.54) and wide income inequalities, such that the top 25% of families earn at least double the population median household income [37]. Based on the local definition of poverty (i.e., <50% of population median income), 1.65 million people, which equals over one fifth of the Hong Kong population, live in poverty [38]. They are eligible for limited financial subsidies (e.g., up to 9488 HKD/month for a family of three in 2012) [39]. Families with monthly household income between 50% and 75% of the population median do not receive much government assistance (e.g., up to 1515 HKD/month for a family of three in 2012) [39]. Tung Chung is a developing district on an outlying island of Hong Kong where around 40% of residents live in poverty [40]. There was only one public primary care clinic in Tung Chung [41] serving 78,000 local residents [42] when this study began in 2012. In 2013, there was a public regional hospital established in Tung Chung. However, it only has primary care, psychiatric, emergency, internal medicine, and allied health services. The public healthcare services in Hong Kong do not provide regular health assessments, and residents have to self-finance for this in the private section. However, Hong Kong tops the world in terms of costs of living, including medical costs [43], and the combination of low-income and limited public healthcare services puts the health of Tung Chung residents at risk.

In 2012, the Trekkers Family Enhancement Scheme (TFES) was initiated by a local philanthropic group, the Kerry Group Kuok Foundation (Hong Kong) Limited (KGKF). The TFES offer supports related to health, education, employment, and environmental harmony to 200 low-income families in Tung Chung. A health empowerment program (HEP) with intercalated annual health assessments, health literacy and self-care enablement courses, and health ambassador training was delivered regularly to support the health of the TFES families. This study aimed to evaluate the 5 year effectiveness of the HEP for the health of low-income families. We examined whether the HEP was associated with greater health enablement, better mental health, and higher health-related quality of life over a 5 year follow-up.

## 2. Materials and Methods

### 2.1. Study Subjects and Data Collection Procedure

The present study was a prospective, comparative cohort study. It involved two groups of low-income families with young children studying in grades 1–3 (aged 7 to 11). All TFES families were invited to enroll in the HEP (herein referred to as “intervention families”), and low-income families who had not participated in the TFES were also recruited in Tung Chung and Kwai Chung as the comparison group. Kwai Chung is also a developing satellite residential district with similar sociodemographics and public healthcare facilities as Tung Chung [44]. Families were recruited between July 2012 and September 2015 if they satisfied all the inclusion criteria: (1) there was at least one family member working (full-time or part-time); (2) there was at least one dependent child in the family who studied in grades 1–3; (3) the monthly income of the family did not exceed 75% of the Hong Kong population median household income; and (4) written consent was provided. Participants in both groups completed a comprehensive health assessment and a telephone questionnaire survey at baseline upon enrollment and at around 5 years after the baseline assessment. All adults and children aged 7–11 years old at the initiation of the study from each family were included in the study.

**Figure 1:**
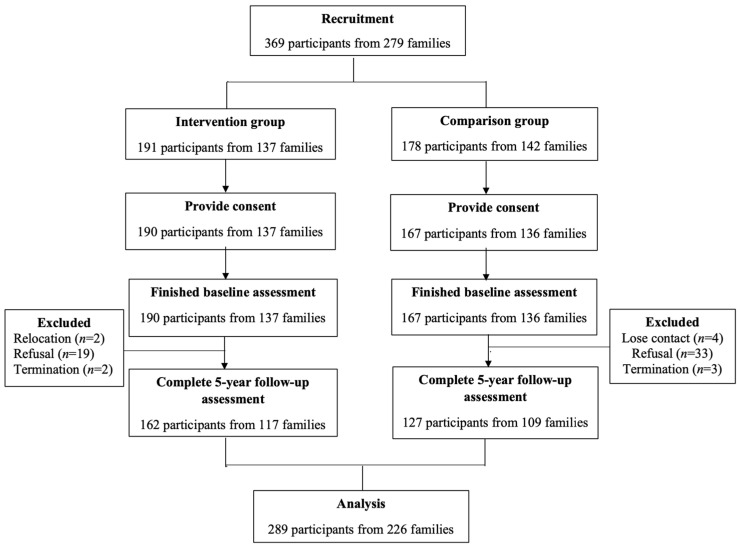
Subject flowchart.

There were 369 adults in total invited from July 2012 to September 2015 (*n* = 191 for the intervention group, *n* = 178 for the comparison group), and 357 adults (*n* = 190 for the intervention group, *n* = 167 for the comparison group) provided consent and completed the baseline assessment. There were 68 participants who did not complete the follow-up assessment at 5 years, representing a drop-out rate of 19% (*n* = 28 for the intervention group, *n* = 40 for the comparison group). Overall, 78% of participants (*n* = 289, *n* = 162 for the intervention group, *n* = 127 for the comparison group) completed the follow-up assessment. These participants were included in the analysis. The mean and median durations of these participants’ follow-ups were both five years. Most of participants were females (72.32%), and their averaged age was 41.63 years old (SD = 6.91). The subject recruitment and follow-up flowchart is presented in Figure 1.

### 2.2. Study Intervention—The HEP

The HEP consisted of intercalated annual health assessments, health talks, self-care enablement courses, and health ambassador training. The health assessment program included an annual telephone health and health service use survey, clinical health assessments, and a health hotline. Based on the telephone surveys and clinical health assessments, those with significant health risks or abnormalities were counseled by a nurse or doctor from the project team or referred to appropriate services for further management. Regular health talks and seminars targeted common problems identified in the health assessments, which included healthy eating, weight management, the health benefits of exercise, liver diseases, nutrition, stress management, psychosomatic illnesses in children, and child development. Self-care enablement courses included stress management, nutrition, dancing and exercise training, and hiking. The courses contained multiple sessions and emphasized participants’ participation. The health talks and enablement courses were all delivered by specialists. The details can be found in Appendix A and Appendix B. During the nutrition and exercise training courses, we encouraged some participants to become the group leaders of the classes and coordinate group practices after class. This group of adults became health ambassadors of their families and peers.

### 2.3. Outcome Measures and Study Instruments

The primary outcome was self-care enablement as assessed by the Chinese version of the Patient Enablement Instrument version 2 (PEI-2), which has been found to be valid and reliable among the local Chinese people [45]. It includes six items on perceived abilities to cope with life, understand and manage illness, maintain health, and help oneself. An example item is, “In the past four weeks, how much have you felt able to cope with life”. Responses to all the items of the PEI were based on a five-point Likert scale, in which 1 meant “not at all” and 5 meant “extremely well”. The scores for each item of the PEI were summated to form the total score, which ranged from 6 to 30. A higher score indicated greater enablement.

The secondary outcomes included negative emotional states and health-related quality of life (HRQOL). We used the Chinese version of the Depression, Anxiety and Stress Scale 21 (DASS-21) to capture participants’ negative emotional states. This measure has shown good reliability in the Chinese setting [46]. DASS-21 includes three seven-item subscales regarding several negative emotional states, including depression, anxiety, and stress. The responses to all the items were provided on a four-point Likert scale, in which 0 meant “did not apply” and 3 meant “very much or most of the time”. An example item is, “I found it hard to wind down”. We first added the scores of the seven items in a subscale and then multiplied the sum by 2 so that they could be compared to the DASS normative data and to other publications on DASS [47]. Each transformed subscale score ranged from 0 to 42. Higher scores indicated greater emotional disturbance.

We utilized the Chinese 12 item Short-Form Health Survey Version 2 (SF-12v2) to measure HRQOL. The Chinese SF-12v2 has shown good validity and reliability among Chinese populations [48,49]. The measure consists of 12 items covering eight domains: physical functioning (PF), general health (GH), bodily pain (BP), physical role-functioning (RP), emotional role-functioning (RE), social functioning (SF), vitality (VT), and mental health (MH). An example item is, “During the past 4 weeks, how much did pain interfere with your normal work (including both work outside the home and housework)?”. These eight domains were weighted into two summary scores: a physical component summary (PCS) score and a mental component summary (MCS) score. The scores for each domain ranged from 0 to 100. The PCS and MCS scores for the SF-12v2 were norm-based. Specifically, the population mean for the two scores was 50 and the standard deviation for the two was 10. A higher score indicated better HRQOL.

Socioeconomic status, general state of health, and physician factors were found to be associated with patient enablement [50]. Given that, confounding factors (covariates), including age, gender, highest education level obtained, household income (monthly), working status, marital status, smoking status, alcohol consumption, obesity status, chronic morbidity, reception of government assistance, and use of a regular family doctor, were collected with a structured questionnaire.

The Chinese PEI-2, DASS-21, SF-12v2, and the covariates questionnaire were administered by a trained interviewer in person or by telephone. All outcome and covariate data were self-reported.

### 2.4. Data Analysis

Data analyses were based on the complete-case analysis and only participants without missing values were included. We used STATA version 16.0 (StataCorp LP, College Station, TX, USA) to conduct all the statistical analyses. The presented tests of significance were two-tailed, with *p* values lower than 0.05 indicating statistical significance.

Descriptive statistics were used to present participants’ baseline characteristics. Inverse probability weighting based on propensity scores was used to account for residual confounding bias and minimize differences in the characteristics of the two groups. We first used a logistic regression model to calculate each participant’s propensity score, with adjustment for the aforementioned baseline covariates. The balance of baseline covariates between the two groups before and after the inverse probability weighting was assessed according to the *p* value, with *p* > 0.05 indicating an optimal balance between two groups.

Cronbach’s α coefficient was measured to test the reliability for internal consistency for each measure, with values larger than 0.7 representing good reliability. Linear regressions weighted by inverse probability weighting using propensity scores were applied to identify the independent effects of the HEP on the participants’ changes in PEI-2, DASS-21, and SF-12v2 scores from baseline to the 5 year follow-up. We first conducted preliminary tests to check the assumptions of the multiple linear regression (e.g., normality, homoscedasticity, and multicollinearity). We checked that there were no cases of outliers (i.e., Cook’s distance < 1 [51]). Although the assumption of normality was violated, it did not substantively affect the results because of the large sample size (i.e., the number of observations per variable was greater than 10 [52]) in the present study. For each model, the *R^2^* and *F*-test of overall significance are reported; the unstandardized coefficients (B), 95% confidence level, and *p*-value are reported to indicate the effects of the intervention on each dependent outcome variable; furthermore, a power analysis for a two-sample means test was applied to calculate the post hoc power. To assess the robustness of the results, we conducted a sensitivity analysis of the multiple linear regressions, adjusting for baseline covariates, without inverse probability weighting.

### 2.5. Ethical Approval

The current study received ethical approval (UW 12–517) from the Institutional Review Board of the University of Hong Kong/Hospital Authority Hong Kong West Cluster.

## 3. Results

Table 1 presents the baseline characteristics of the subjects by group before and after weighting. Before weighting, the marital and working statuses of the two groups were significantly different. Nevertheless, all baseline characteristics were balanced after propensity score weighting. In the weighted sample, most subjects were between 35 and 50 years old (72.79%), and they were predominantly women (71.26%).

The measures showed good reliability with the data from the current study, including the PEI-2 (Cronbach’s α = 0.90), DASS-21 (Cronbach’s α = 0.84 for depression, Cronbach’s α = 0.79 for anxiety, Cronbach’s α = 0.85 for stress), and SF-12v2 (Cronbach’s α = 0.73 for PCS, Cronbach’s α = 0.81 for MCS). The PEI-2, DASS-21, and SF-12v2 scores at the baseline and follow-up assessments before and after weighting are presented in Table 2 for both the intervention and comparison groups.

Table 3 presents the results for the regression of the HEP’s impact on the 5 year changes in the PEI-2, DASS-12, and SF-12v2 scores after propensity score weighting. The HEP intervention group showed significantly greater increases in all the items and the total score for the PEI-2 (B ranged between 0.59 and 5.22, all *p* < 0.001). Additionally, the HEP was significantly associated with a greater decrease in the DASS depression score (B = −1.98, *p* = 0.001). Nevertheless, the differences in the changes in the DASS anxiety and stress scores between the two groups were not significant. There was a positive association between the HEP and increases in MCS scores (B = 2.99, *p* = 0.027), but there was no difference in the changes in PCS scores between groups. The results consistently showed significantly greater increases in all the items and the total score for the PEI-2 in the HEP group than in the comparison group in the sensitivity analysis using multiple linear regression to adjust for baseline covariates (Appendix C).

## 4. Discussion

As far as we know, the current longitudinal study is the first to examine the long-term health effects of a complex HE intervention in low-income families. The results with inverse probability weighting showed that participants in the HEP demonstrated greater increases in self-care enablement (PEI-2 scores) and mental HRQOL (SF-12v2 MCS score) and greater decreases in depressive symptoms (DASS depression score) than those without involvement in the HEP intervention.

Previous studies have demonstrated the effectiveness of various HEPs in improving participants’ health-related outcomes, but most of them were short-term studies lasting for only a few months with follow ups of less than one year [53]. In particular, studies have shown that involvement in a short-term HEP can modify health-related attitudes [13,14,15,16]. For example, an HEP with a combination of teaching sessions, discussions, role playing, field tours, and so forth enhanced self-efficacy in self-care for 94 community-dwelling older adults in Taiwan after 12 weeks [16]. A HEP with 5 month problem-based learning sessions increased the perceived health control and sense of capacity to take health action among 63 immigrant women in Taiwan [14]. Furthermore, HEPs can improve psychological health [12,19]. For instance, by delivering a series of education courses over four months, an HEP increased psychological well-being and health-related quality of life for 54 Italians with diabetes [54]. An HEP with six weekly home visits by a trained nurse was found to improve psychological well-being for 32 homebound older adults in the United States [17]. As indicated above, although most HEPs only intervene for a few months and focus on small-scale individual programs, the effectiveness of these HEPs has been demonstrated across various populations, including for different ages [15,16,17,19], genders [14], ethnic groups [18], immigration statuses [14], health statuses [13,15,55], and regions [16,56]. However, research on the long-term effectiveness of HEPs and whether a family-based complex intervention is feasible and applicable in low-income families is limited.

The findings of the present study complement those of previous studies in affirming the long-term effectiveness of HEPs in improving participants’ self-care enablement and mental health. One explanation regarding the mechanism of how HEPs increase enablement and mental health is that regular health assessments engage participants and raise their health awareness [57]; the health talks, self-care enablement courses, and advice on appropriate management empower them to take control of their health and cope with health problems [58]. This sense of control has the potential to inhibit the triggering of negative emotions and help with their management [17], therefore increasing the MCS score and decreasing the DASS depression score. The results indicate the feasibility and applicability of HEPs with interrelated components for the improvement of health care enablement and self-reported mental health among parents in low-income families. In particular, Hong Kong is one of the regions that has the highest Gini coefficients and significant income inequity [37]. The effectiveness of HEPs in Hong Kong targeting low-income families suggests the possibility of using HEPs to reduce health equity.

Additionally, previous studies have indicated that HEPs can stimulate the adoption of healthy habits. For example, a Korean study showed that utilizing 8 week lifestyle improvement education, group discussions, and exercise training enabled 27 hypertensive older adults to increase physical activity and reduce sedentary behavior [15]. In contrast, we did not find any significant association between the HEP and increases in physical HRQOL (SF-12v2 PCS score). In general, we observed a decrease in the PCS scores in both groups over the 5 year follow up, which coincided with the outbreak of the COVID-19 pandemic. During the peak of the COVID-19 outbreak, the Hong Kong government released mandates to close indoor sports facilities and limit outdoor activities for over five months in 2020 [59]. The outbreak of the COVID-19 pandemic and limited physical activities could have reduced physical fitness, leading to worsening of physical functioning and general health, which are the major determinants of the SF-12v2 PCS [60]. This suggests that the failure of the current HEP to improve SF-12v2 PCS may have been related to the outbreak of the COVID-19 pandemic and its influence on individuals’ physical activity and physical health. Additionally, during the pandemic, the formats of the intervention components in the current HEP changed because the face-to-face intervention was not feasible. We therefore delivered the intervention via real-time video meetings, distribution of videos, a health app, telephone consultations, and so forth. While the hybrid mode of the HEP was successfully in improving self-reported self-enablement and mental health, the nonsignificant association between the HEP and the SF-12v2 PCS score suggested that the effect of the hybrid mode on physical health may have been limited.

The comparison between the current HEP and other HE interventions involving low-income families [24,25,26,27,28,29,30,31] showed two main differences in terms of intervention design. First, our HEP included four interrelated components that covered various aspects of health by providing health knowledge, encouraging regular exercise, promoting family activities, facilitating regular health assessments and cues to take health action, increasing social interaction, and offering health consultations. Second, the complex intervention involved whole families, including children and parents. The association between family members’ health was considered in the design of the intervention components to maximize the effectiveness of the HEP. The involvement of the whole family and various components likely increased participation and participants were seldom lost during follow up, which also indicate the success of the present intervention.

The findings of the current intervention contribute to the current health empowerment literature in three key ways. First, health empowerment among low-income families is feasible and has sustainable effectiveness. Second, both adults and children should be involved in the regular health assessment and self-care enablement activities in light of the close links among family members’ health and the mutual influence on health-seeking behavior. Third, multi-dimensional but intercalated components can be utilized to enhance not only health promotion knowledge and practice but also accessible professional support to solve health problems. The various components provided insights into the design of future complex health interventions in real-world settings.

There are four potential limitations worthy of further discussion. First, participants in the HEP were those who volunteered to join and may therefore have had a prior interest in receiving health information and resources. Additionally, the intervention was limited to people who were currently residing in one district in Hong Kong, so the sample cannot be viewed as representative. Non-randomization and district limitations may affect the generalizability of the findings and their external validity. Second, since it was not a randomized control trial, the observed and unobserved confounding variables could not be fully accounted for in the analysis. However, our main analysis adjusted for residual confounding by applying inverse probability weighting based on propensity score. Third, we used face-to-face or telephone interviews to collect data. Furthermore, data regarding the variables of interest were based on participants’ self-reports. The different data collection methods and the self-reported data could have had the potential to cause measurement errors, but this was equally applicable to both the intervention and comparison groups. Finally, the long recruitment period and the loss of participants during follow up could have led to bias in the results. Nevertheless, the drop-out rates in this cohort study in both groups were lower than the upper limit of the acceptable rate (i.e., 50%) [61], which supported the reliability and validity of the results.

## 5. Conclusions

This study shows the effectiveness of a longitudinal HEP in enabling self-care and improving mental health among adults from low-income families. It offers insights into the feasibility and applicability of HEPs in real-world community settings. Given the increasingly wide income inequality and the close link between poverty and poor health, similar HEPs may help to break the vicious cycle. This opens a new research agenda regarding how HEP care models can be more widely implemented to enhance health equity.

## Figures and Tables

**Table 1:** Baseline characteristics before and after applying inverse probability weighting using propensity score.

	Before Weighting				After Weighting			
	Total (*n* = 289)	Intervention Group (*n* = 162)	Comparison Group (*n* = 127)	*p*-Value	Total	Intervention Group	Comparison Group	*p*-Value
Age (%), year	41.63 ± 6.91	41.10 ± 7.01	42.31 ± 6.74	0.140	41.74 ± 7.57	41.03 ± 7.20	42.43 ± 0.68	0.114
<35	17.99%	19.14%	16.54%	0.831	17.43%	18.03%	16.84%	0.933
≥35 and <50	72.32%	70.99%	74.02%		72.79%	72.80%	72.78%	
≥50	9.69%	9.88%	9.45%		9.78%	9.17%	10.38%	
Gender (%, *n*)				0.173				0.959
Female	72.32%	69.14%	76.38%		71.26%	71.41%	71.11%	
Male	27.68%	30.86%	23.62%		28.74%	28.59%	28.89%	
Educational level (%)				0.271				0.923
No formal education/primary	14.19 %	16.67%	11.02%		12.95%	13.75%	12.16%	
Secondary	79.58 %	78.40%	81.10%		80.88%	80.36%	81.39%	
Tertiary/further education	6.23 %	4.94%	7.87%		6.17%	5.89%	6.45%	
Working status (%)				<0.001 *				0.960
Working (employee or employer)	73.36%	81.48%	62.99%		73.84%	73.98%	73.70%	
Not working (homemaker, retired, unemployed)	26.64%	18.52%	37.01%		26.16%	26.02%	26.30%	
Marital status (%)				0.020 *				0.829
Married	85.12%	90.12%	78.74%		85.50%	85.71%	85.29%	
Divorced	10.38%	8.02%	13.39%		10.88%	11.38%	10.39%	
Unmarried (single/widower)	4.50%	1.85%	7.87%		3.62%	2.92%	4.32%	
Monthly family income ^#^ (%), HKD				0.344				0.910
≤11,000	39.45%	37.04%	42.52%		39.71%	40.06%	39.36%	
>11,000	60.55%	62.96%	57.48%		60.29%	59.94%	60.64%	
Government assistance (%)				0.076				0.769
Yes	20.42%	16.67%	25.20%		21.73%	20.91%	22.53%	
No	79.58%	83.33%	74.80%		78.27%	79.09%	77.47%	
Obesity status (%)				0.996				0.980
Normal weight	58.13%	58.02%	58.27%		58.49%	58.84%	58.14%	
Overweight	37.02%	37.04%	37.01%		35.99%	35.95%	36.03%	
Obese	4.84%	4.94%	4.72%		5.52%	5.21%	5.83%	
Chronic morbidity (%)				0.236				0.817
Yes	42.56 %	60.49%	53.54%		41.52%	40.79%	42.23%	
No	57.44 %	39.51%	46.46%		58.48%	59.21%	57.77%	
Smoking status (%)				0.907				0.999
Never smoked	77.85%	77.16%	78.74%		75.52%	75.66%	75.38%	
Quit	6.92%	6.79%	7.09%		7.53%	7.45%	7.61%	
Current smoker	15.22%	16.05%	14.17%		16.95%	16.88%	17.02%	
Alcohol consumption (%)				0.125				0.986
Never drank	69.20%	72.84%	64.57%		68.84%	69.24%	68.45%	
Quit	3.46%	4.32%	2.36%		3.93%	3.73%	4.13%	
Current drinker	27.34%	22.84%	33.07%		27.23%	27.03%	27.42%	
Use of a regular family doctor (%)				0.694				0.746
Yes	15.92%	16.67%	14.96%		15.04%	15.76%	14.34%	
No	84.08%	83.33%	85.04%		84.96%	84.24%	85.66%	

Note. The total % may not add up to 100% because of rounding; * *p* values < 0.05 were statistically significant; ^#^ the averaged HK population median monthly household income in 2012–2015 was around HKD 22,000; statistical difference was tested with an independent samples *t*-test or Chi-squared test whenever appropriate.

**Table 2:** Comparison of changes in PEI-2, DASS-21, and SF-12 for intervention and comparison groups.

	Before Weighting				After Weighting			
	Baseline		Follow-Up		Baseline		Follow-Up	
	Intervention Group	Comparison Group	Intervention Group	Comparison Group	Intervention Group	Comparison Group	Intervention Group	Comparison Group
	Mean ± SD	Mean ± SD	Mean ± SD	Mean ± SD	Mean ± SD	Mean ± SD	Mean ± SD	Mean ± SD
PEI-2	*n* = 162	*n* = 127	*n* = 162	*n* = 127				
1. Able to cope with life	2.84 ± 1.00	4.02 ± 0.89	3.79 ± 0.97	3.89 ± 1.02	2.83 ± 0.94	4.05 ± 0.93	3.79 ± 0.97	3.88 ± 1.08
2. Able to understand your illness	2.96 ± 0.88	3.62 ± 0.92	3.55 ± 0.89	3.61 ± 0.90	2.94 ± 0.84	3.55 ± 1.00	3.56 ± 0.87	3.58 ± 0.96
3. Able to cope with your illness	2.84 ± 1.01	3.72 ± 0.88	3.58 ± 0.89	3.65 ± 0.95	2.81 ± 0.98	3.65 ± 0.96	3.57 ± 0.88	3.58 ± 0.99
4. Able to keep yourself healthy	2.87 ± 0.99	3.61 ± 0.80	3.49 ± 0.89	3.57 ± 0.87	2.83 ± 0.95	3.58 ± 0.86	3.47 ± 0.88	3.55 ± 0.87
5. Confident about your health	2.78 ± 0.97	3.57 ± 0.82	3.46 ± 0.94	3.57 ± 0.81	2.76 ± 0.94	3.58 ± 0.83	3.42 ± 0.95	3.59 ± 0.80
6. Able to help yourself	2.61 ± 0.97	4.11 ± 0.77	3.86 ± 0.93	3.98 ± 0.85	2.62 ± 0.93	4.11 ± 0.79	3.85 ± 0.93	4.00 ± 0.84
Total PEI-2 score	16.90 ± 4.65	22.65 ± 3.63	21.73 ± 4.49	22.28 ± 4.13	16.78 ± 4.44	22.52 ± 3.81	21.66 ± 4.46	22.17 ± 4.23
DASS-21	*n* = 151	*n* = 130	*n* = 151	*n* = 130				
Depression score	4.28 ± 5.72	4.32 ± 6.52	3.47 ± 5.22	5.43 ± 6.54	4.30 ± 5.72	4.07 ± 6.16	3.51 ± 5.23	5.26 ± 6.22
Anxiety score	4.85 ± 6.00	4.71 ± 5.44	4.78 ± 5.80	5.54 ± 6.03	4.76 ± 5.98	4.54 ± 5.14	4.76 ± 5.81	5.51 ± 5.88
Stress score	7.50 ± 7.84	7.93 ± 7.64	6.10 ± 7.02	7.87 ± 6.99	7.50 ± 7.94	7.63 ± 7.37	6.06 ± 7.00	7.63 ± 6.60
SF-12V2	*n* = 175	*n* = 149	*n* = 175	*n* = 149				
PCS	48.80 ± 8.45	49.84 ± 7.55	48.08 ± 9.03	49.63 ± 8.24	48.76 ± 8.67	49.57 ± 7.79	47.72 ± 9.06	49.87 ± 8.20
MCS	50.05 ± 11.43	51.64 ± 11.16	53.32 ± 10.23	52.77 ± 8.56	49.24 ± 11.62	52.04 ± 11.16	53.37 ± 10.29	53.18 ± 8.66

Note. PEI-2 = Patient Enablement Instrument version 2; DASS-21= Depression, Anxiety and Stress Scale 21; SF-12v2 = 12 item Short-Form Health Survey version 2; PCS = physical component summary score; MCS = mental component summary score; SD = standard deviation.

**Table 3:** Association between health empowerment program participation and changes in outcomes at 5 year follow-up after propensity score weighting.

	B (95% CI)	*p*-Value for B	*R* ^2^	*F*	Significance for *F* Test	Post Hoc Power
PEI-2						
1. Able to cope with life	1.13 (0.79, 1.47)	<0.001 *	0.15	43.11	<0.001 *	1.00
2. Able to understand your illness	0.59 (0.27, 0.90)	<0.001 *	0.05	13.45	<0.001 *	0.92
3. Able to cope with your illness	0.83 (0.52, 1.15)	<0.001 *	0.10	26.55	<0.001 *	1.00
4. Able to keep yourself healthy	0.67 (0.37, 0.97)	<0.001 *	0.07	19.81	<0.001 *	0.99
5. Confident about your health	0.66 (0.35, 0.97)	<0.001 *	0.06	17.51	<0.001 *	0.98
6. Able to help yourself	1.34 (1.02, 1.66)	<0.001 *	0.22	69.09	<0.001 *	1.00
Total PEI-2 Score	5.22 (3.81, 6.63)	<0.001 *	0.17	53.17	<0.001 *	1.00
DASS-21						
Depression score	−1.98 (−3.48, −0.47)	0.001 *	0.03	6.72	0.010 *	0.66
Anxiety score	−0.96 (−2.42, 0.49)	0.193	0.01	1.70	0.193	0.23
Stress score	−1.44 (−3.31, 0.42)	0.129	0.01	2.32	0.129	0.29
SF-12V2						
PCS	−1.34 (−3.82, 1.14)	0.290	0.00	1.12	0.290	0.16
MCS	2.99 (0.34, 5.64)	0.027 *	0.02	4.94	0.027 *	0.55

Note: PEI-2 = Patient Enablement Instrument version 2; DASS-21 = Depression, Anxiety and Stress Scale 21; SF-12v2 = 12 item Short-Form Health Survey version 2; PCS = physical component summary score; MCS = mental component summary score; * *p* values < 0.05 were statistically significant.

## Data Availability

Data from this study are available upon request.

## Appendix A

**Table A1:** Health literacy program.

	Date	Content
2012–2013	26 May 2012	Cough and common colds, common eye conditions
	4 August 2012	Child development/ADHD
	6 October 2012	Psychosomatic illness in children, stress related to parenting
	1 December 2012	Chinese medicine and yangsheng
2013–2014	30 November 2013	Oral health, oral hygiene practice and common oral/dental problems, weight management, healthy cooking techniques, and food selection
2014–2015	2 March 2014	Qigong
	24 May 2014	Ergonomics, exercise, and fitness training help to protect from back pain
	5 July 2014	Liver disease management—liver disease
	13 December 2014	Body constitutional types in Chinese medicine
2015–2016	11 April 2015	Common eating problems
	9 May 2015	Introduce my plate
	13 June 2015	Fat facts: know more about oil
	18 July 2015	Summary and nutrition Q&A
	5 December 2015	Body constitutional types in Chinese medicine
	5 December 2015	Common female health problems
2016–2017	9 April 2016	Knowing my plate
	16 April 2016	Tasting event—try new foods
	23 April 2016	Supermarket field trip—label reading
	30 April 2016	Family cooking
	17 December 2016	Men’s body constitutional types in Chinese medicine
	17 December 2016	Men’s health problems
2017–2018	22 April 2017	Knowing your nutrition needs
	29 April 2017	How to value food
	6 May 2017	Recipe revamp
	13 May 2017	Be a smart eater
	4 November 2017	Sexual health of teenagers
	4 November 2017	Positive psychology
2018–2019	5 May 2018	Knowing my signature dish
	15 May 2018	Turning old to new
	26 May 2018	Adding color to your dish
	2 June 2018	Menu of the week
	1 December 2018	Understand the causes, screening for H. Pylori, other clinical assessments, management using Western medication—from the view of Western medicine
	1 December 2018	Understand the causes, management for different body constitutions—from the view of Chinese medicine
2019–2020	4 May 2019	Healthy home cooking—foods for chronic disease prevention
	11 May 2019	Healthy home cooking—recommendations for festive dishes
	18 May 2019	Healthy home cooking—food recommendations for children and older adults
	25 May 2019	Healthy home cooking —foods for skin care and improving immune system
	30 August 2019	Nutrition talk—understanding the concept of “fruits and vegetables 2 + 3 a day” and tips for practice
	25 October 2019	Dental talk—focus on understanding dental problems and methods of prevention
2020–2021	1 August 2020	Healthy home cooking—introduction to the concept of the “three lows”
	8 August 2020	Healthy home cooking —sodium in food/condiments and use of low-sodium alternatives in cooking
	15 August 2020	Healthy home cooking —identification of healthier cooking methods and ingredients
	22 August 2020	Healthy home cooking—Recognizing hidden sugar in food and knowing how to cut down on sugar intake or find alternatives when craving
	9 January 2021	Stress management talk
	20 February 2021	Visual health talk

## Appendix B

**Table A2:** Self-care enablement program.

	Date	Content
2012–2013	9 November 2012–19 December 2012 (6 sessions)	Healthy living and healthy family
2013–2014	10 April 2013–22 May 2013 (6 sessions)	Mental health enhancement
2014–2015	26 April 2014–14 June 2014 (8 sessions)	Eight-section exercise 1
	19 July 2014–13 September 2014 (8 sessions)	Eight-section exercise 2
	1 November 2014–20 December 2014 (6 sessions)	Tai chi
	1 January 2014–31 July 2014 (15 sessions)	Family well-being enablement counseling
	1 May 2014–30 August 2014 (2 sessions)	Family well-being workshops
2015–2016	30 May 2015–18 July 2015 (8 sessions)	Eight-section exercise
	28 March 2015–16 May 2015 (8 sessions)	Mawangdui daoyin exercises
	10 October 2015–16 January 2016 (8 sessions)	Physiotherapy class
2016–2017	12 March 2016 (1 session)	Nutrition education for children
	9 April 2016–30 April 2016 (4 sessions)	Nutrition workshop
	16 July 2016–6 August 2016 (4 sessions)	Kids and parents learn healthy recipes together
	9 July 2016–30 July 2016 (4 sessions)	Boxing class for teenagers
	4 August 2016–30 August 2016 (4 sessions)	Dancing class for teenagers
	8 October 2016–3 December 2016 (8 sessions)	Parents exercise together with their children
2017–2018	22 April 2017–13 May 2017 (4 sessions)	Kids and parents learn healthy recipes together
	3 March 2017–24 March 2017 (4 sessions)	Rope skipping class 1
	3 August 2017–10 August 2017 (4 sessions)	Rope skipping class 2
	11 November 2017–23 December 2017 (4 sessions)	Rope skipping class 3
	14 September 2017–21 October 2017 (6 sessions)	Dancing gym
2018–2019	5 May 2018–2 June 2018 (4 sessions)	Kids and parents learn healthy recipes together
	25 April 2018–12 September 2018 (6 sessions)	Dancing gym
	19 September 2018–23 October 2018 (6 sessions)	Dancing gym 2
	20 February 2018–27 March 2018 (6 sessions)	Dancing gym 3
	19 July 2018–16 August 2018 (5 sessions)	Rope skipping class 1
	10 November 2018–26 January 2019 (6 sessions)	Rope skipping class 2
2019–2020	4 May 2019–25 May 2019 (4 sessions)	Kids and parents learn healthy recipes together
	29 May 2019–3 July 2019 (6 sessions)	Dancing gym 1
	11 September 2019–16 October 2019 (6 sessions)	Dancing gym 2
	30 July 2019 (1 session)	Summer sports
	2 November 2019–30 November 2019 (4 sessions)	Walking group
2020–2021	1 August 2020–22 August 2020 (4 sessions)	Kids and parents learn healthy recipes together
	27 August 2020–31 August 2020 (6 sessions)	Dancing gym 1
	16 December 2020–3 March 2020 (8 sessions)	Dancing gym 2
	10 March 2021–31 March 2021 (5 sessions)	Dancing gym 3

## Appendix C

**Table A3:** Associations between health empowerment program participation and changes in outcomes at 5 year follow-up before propensity score weighting.

	B (95% CI)	*p*-Value for B	*R^2^*	*F*	Significance for *F* Test	Post Hoc Power
PEI-2						
1. Able to cope with life	1.12 (0.77, 1.47)	<0.001 *	0.17	2.89	<0.001 *	1.00
2. Able to understand your illness	0.66 (0.35, 0.96)	<0.001 *	0.13	2.07	0.006 *	0.99
3. Able to cope with your illness	0.87 (0.54, 1.19)	<0.001 *	0.13	2.18	0.004 *	1.00
4. Able to keep yourself healthy	0.68 (0.36, 0.99)	<0.001 *	0.11	1.79	0.023 *	1.00
5. Confident about your health	0.65 (0.33, 0.98)	<0.001 *	0.12	2.02	0.008 *	1.00
6. Able to help yourself	1.37 (1.04, 1.69)	<0.001 *	0.25	4.68	<0.001 *	1.00
Total PEI-2 score	5.35 (3.81, 6.89)	<0.001 *	0.18	3.21	<0.001 *	1.00
DASS-21						
Depression score	−2.01 (−3.68, −0.35)	0.02	0.08	1.06	0.392	0.69
Anxiety score	−0.85 (−2.45, 0.76)	0.300	0.06	0.82	0.684	0.23
Stress score	−1.50 (−3.56, 0.57)	0.154	0.05	0.66	0.853	0.29
SF-12V2						
PCS	−1.24 (−3.69, 1.21)	0.321	0.05	0.75	0.767	0.07
MCS	3.15 (0.28, 6.02)	0.031 *	0.08	1.21	0.246	0.35

Note: PEI-2 = Patient Enablement Instrument version 2; DASS-21 = Depression, Anxiety and Stress Scale 21; SF-12v2 = 12 item Short-Form Health Survey version 2; PCS = physical component summary score; MCS = mental component summary score; regression models adjusted for gender, age, education level, family income, marriage status, working status, chronic disease, drink status, smoke status, obesity status, reception of government assistance, and whether participants were registered with a family doctor. * *p* values < 0.05 were statistically significant.
